# The Complete Mitochondrial Genome of the *Luciocyprinus langsoni* (Cypriniformes: Cyprinidae): Characterization, Phylogeny, and Genetic Diversity Analysis

**DOI:** 10.3390/genes15121621

**Published:** 2024-12-18

**Authors:** Tiezhu Yang, Chenxi Tan, Liangjie Zhao, Zhiguo Hu, Chaoqun Su, Fan Li, Yuanye Ma, Wenchao Zhang, Xiaoyu Hao, Wenxu Zou, Jiayin Kang, Qingqing He

**Affiliations:** 1 School of Fisheries, Xinyang Agriculture and Forestry University, Xinyang 464000, China; yangtiezhu1234@163.com (T.Y.); tanchenxi2013@163.com (C.T.); a850924t@163.com (L.Z.); 18508927582@163.com (Z.H.); aurora15738591913@163.com (W.Z.); hao1873678@163.com (X.H.); zwx0552@163.com (W.Z.); kjy040610@163.com (J.K.); xqxq0628@163.com (Q.H.); 2 Fishery Biological Engineering Technology Research Center of Henan Province, Xinyang 464000, China; 3 Shanghai Natural History Museum, Branch of Shanghai Science and Technology Museum, Shanghai 200041, China; 4 Xinyang Nanwan Reservoir Fishery Development Co., Ltd., Xinyang 464000, China; yuanye19872372@126.com

**Keywords:** *Luciocyprinus langsoni*, mitochondrial genome, phylogenetic analysis, genetic diversity

## Abstract

**Background**: *Luciocyprinus langsoni* is a species belonging to the Cyprinidae family. The objective of this study is to gain a comprehensive understanding of its evolutionary history and genetic characteristics. **Methods**: The complete mitochondrial genome of *L*. *langsoni* was determined using overlapping PCR. A phylogenetic analysis was conducted based on 13 protein-coding genes from 48 species. A population genetic diversity analysis using the *COI* gene and a selection analysis of 13 protein-coding genes were also performed. **Results**: The mitogenome is 16,586 base pairs long and consists of 13 protein-coding genes, two ribosomal RNAs, 22 transfer RNAs, and two control regions. It has a high adenine-thymine (A + T) content. The phylogenetic analysis confirms the placement of *L*. *langsoni* within the subfamily *Cyprininae*. The population genetic diversity analysis reveals low variability in the Hechi Longjiang population. The selection analysis shows that all 13 protein-coding genes have evolved under purifying selection with Ka/Ks ratios below 1. **Conclusions**: These results enhance our understanding of *L*. *langsoni*’s evolutionary history and lay a genetic foundation for future studies in population genetics and phylogenetics.

## 1. Introduction

The mitochondrial genome (mitogenome) serves as a critical molecular marker in evolutionary and population genetics, characterized by unique properties distinct from nuclear DNA [1,2]. This fish mitogenome features a compact, circular structure, typically 15,000 to 18,000 base pairs long, composed of heavy and light strands, with transcription primarily on the heavy strand [3,4]. It encodes for a suite of essential genetic elements, including 13 protein-coding genes (PCGs), two ribosomal RNA genes (*12S rRNA* and *16S rRNA*), 22 transfer RNA genes (tRNAs), and a control region that contains regulatory elements vital for transcription and replication [4]. Being capable of adopting a conserved secondary structure, the control region encompasses the origins of replication and transcription, thus highlighting its vital role in maintaining mitochondrial DNA (mtDNA) [5]. Mitochondrial DNA is distinguished by its matrilineal inheritance, rapid evolutionary pace, and minimal intermolecular recombination, making it an effective tool for delineating population genetic characteristics and conducting phylogenetic analyses [6]. Although mtDNA fails to capture the genetic information of the male population [7,8], its conservation in amino acid coding, coupled with other attributes, has established its utility in molecular evolution, phylogenetics, and comparative genomics [9]. Furthermore, the mitogenome’s tRNA molecular structure, gene arrangement, and models of replication-transcription control have been pivotal in facilitating deep-level phylogenetic inferences within taxonomy [10]. The sequencing and annotation of numerous fish mitogenomes have significantly broadened our comprehension of their phylogeny, biogeography, and population genetic structure [4]. Given the mitogenome’s simplicity, small size, and high copy number, it remains an indispensable resource in molecular biology and genetic research [3].

The genus *Luciocyprinus*, despite its morphological and behavioral similarities to the genus *Elopichthys*, is taxonomically distinct. The subfamily affiliation of *Luciocyprinus* has been a subject of debate. In 1933, Lin classified it into the subfamily Gobioninae based on the characteristic of having two rows of pharyngeal teeth [11]. Studies on skeletal structure by Wu (1977) and Chen (1984) led to its classification into the subfamily *Barbinae* [12,13]. However, Lei (2015), based on phylogenetic and polyploidy research, assigned *Luciocyprinus* to the subfamily *Cyprininae* [14,15]. Despite these classifications, some scholars still advocate for its placement within the subfamily *Barbinae* based on morphological considerations. The genus *Luciocyprinus* includes two reported species: *L. langsoni* (Vaillant, 1904) and *Luciocyprinus striolatus* (Cui and Chu, 1986) [16]. Due to extensive habitat destruction caused by industrial development and overfishing, the populations of both species have sharply declined, with local extinctions occurring in some areas [17]. Both species were rated as Vulnerable (VU) by the IUCN in 2012 [18] and listed as second-class protected animals in the 2021 “National Key Protected Wildlife List” [19]. *L. langsoni* is a predatory fish inhabiting the upper-middle water layers, recognized for its strong swimming ability and proactive hunting behavior. It typically resides in rivers and lakes with certain water flow speeds, while juveniles under 16 cm in length prefer slower-flowing bays or still waters. This species is primarily distributed in the Xijiang River system within the Pearl River basin. Given its delicious flesh, large size, and rapid growth, *L. langsoni* holds significant economic value if artificial breeding can be successfully achieved.

In this study, we sequenced, assembled, and characterized the complete mitochondrial genome of *L. langsoni*, examining its size, structure, organization, nucleotide composition, codon usage, tRNA molecular structures, and evolutionary rates. We also assessed the population genetic diversity of *L. langsoni* using the *COI* gene, which is essential for elucidating genetic variation among and within populations. Integrating our data with NCBI database information, we delineated the phylogenetic position of *L. langsoni* within the genus *Luciocyprinus* and subfamily *Cyprininae*, utilizing 13 PCGs. Our results establish a foundational dataset for future phylogenetic and taxonomic research on *Luciocyprinus*, illuminating the mitochondrial genome features and evolutionary relationships of this species.

## 2. Materials and Methods

### 2.1. Sample Collection, DNA Isolation, PCR Amplification, and Sequencing

Adult specimens of *L. langsoni* were collected from the Longjiang River, Hechi City, Guangxi Province, China (24°30′ N, 108°37′ E). Our procedures followed international guidelines for laboratory animal care and treatment. Collected samples were preserved in 100% ethanol and stored at −80 °C until DNA extraction (voucher number: XYAFU-Mo-s190511630). Total DNA was extracted from muscle tissue using the Magnetic Animal Tissue Genomic DNA Kit (Tiangen Biotech Co., Beijing, China). DNA quality was assessed by 1.5% agarose gel electrophoresis, and the DNA was kept at −20 °C until PCR amplification of the entire mitogenomes. The complete mitochondrial genome of *L. striolatus* (GenBank accession number: AP012525) was used as a template for primer design. A total of 12 pairs of primers were designed based on their conserved regions for amplification by overlapping PCR (Table 1). The PCR amplification experiment was carried out using the LA Taq DNA polymerase reagent (Takara, Beijing, China) in a 50-microliter system. The system contents include 25 microliters of 2 × LA Taq Premix buffer, 2 microliters of template DNA, 1.5 microliters each of upstream and downstream primers (10 nM), and 20 microliters of deionized water. The PCR amplification program was set as follows: initial denaturation of DNA at 94 °C for 3 min, followed by 35 cycles of denaturation at 94 °C for 30 s, annealing at 49.9–58.2 °C (Table 1) for 30 s, and extension at 72 °C for 2 min. Finally, there is a long extension at 72 °C for 10 min to end the program. Amplified PCR products were verified on 1% agarose gels, and their sizes were estimated against a DNA Marker S (100–5000 bp) (Sangon Biotech, Shanghai, China). The PCR products were subjected to bidirectional sequencing using the same primers on a 3730xl DNA analyzer (Applied Biosystems, Thermo Fisher Scientific, Waltham, MA, USA) at Sangon Biotech, Shanghai, China, achieving 100% coverage of the amplified regions [20].

### 2.2. Sequence Assembly, Annotation, and Bioinformatics Analysis

The sequencing results of 12 amplified fragments were assembled into a circular sequence by using the SeqMan software (v 7.1.0) in the DNA STAR package (DNAStar Inc., Madison, WI, USA). The mitochondrial genome sequence annotation was performed automatically with the Galaxy Web Server’s MITOS2 de novo annotation tool [21]. tRNA genes were identified and annotated using the tRNAscan-SE 2.0 search server [22]. The base composition and codon usage of the mitochondrial genome of *L. langsoni* were analyzed using MEGA 11.0 software [23], while the relative synonymous codon usage (RSCU) for each protein-coding gene (PCG) was analyzed using CodonW 1.4.2 [24]. The A + T skew value was calculated using the formula A + T skew = (A% − T%)/(A% + T%), and the G + C skews was obtained by calculation using formula G + C skew = (G% − C%)/(G% + C%), respectively [25]. KaKs Calculator 3.0 [26] was employed to determine the substitution rates, including Ka (nonsynonymous substitution rate) and Ks (synonymous substitution rate), among closely related species.

### 2.3. Phylogenetic Analyses

A phylogenetic analysis encompassing 48 Cyprinidae species across 18 genera was conducted. AliView 1.2.6 software [27] facilitated the alignment of amino acid sequences derived from 13 PCGs. Mitogenome sequences for these 48 species were sourced from GenBank (Table 2). *Sinogastromyzon szechuanensis* (Fang, 1930) and *Myxocyprinus asiaticus* (Bleeker, 1864) were designated as outgroups [28]. PartitionFinder 2.1.1 identified the most suitable evolutionary models for our analysis [29]. After arranging and splicing the nucleotide sequences of 13 PCGs in a specific order, the maximum likelihood method (ML) and Bayesian inference method (BI) were used, respectively, to analyze the phylogenetic relationships. The ML tree was generated with IQ-TREE v. 2.3.6 [30], employing 1000 bootstrap replicates. MrBayes v. 3.2.7a [31] executed the BI analysis, with four Markov chain Monte Carlo (MCMC) chains running for 2 million generations, sampling every 1000. A burn-in of 25% was applied to the initial sampled data. FigTree v1.4.4 [32] was utilized for the generation and visualization of phylogenetic trees.

### 2.4. Population Genetic Diversity Analysis

In this research, we procured DNA samples from 30 adult specimens of *L. langsoni* and stored them. For assessing population genetic diversity, we finally selected the *COI* gene located on the mitochondrial genome of *L. langsoni*. We designed specific primers for *COI* gene amplification: LL-COI-F (5′-CCAGCGAGCATTCATCTACT-3′) and LL-COI-R (5′-AACCTGCGATTTCACCTTG-3′), aimed at amplifying an 1800 bp fragment. Subsequent bidirectional sequencing of the PCR products was performed by Shanghai Sangon Biotech, and the sequences were assembled using DNAstar software (v 7.1.0). The analysis of genetic diversity was then carried out using SeqMan software (v 7.1.0, DNAStar Inc., Madison, WI, USA).

## 3. Results and Discussion

### 3.1. Mitogenome Genome Organization of L. langsoni

**Figure 1:**
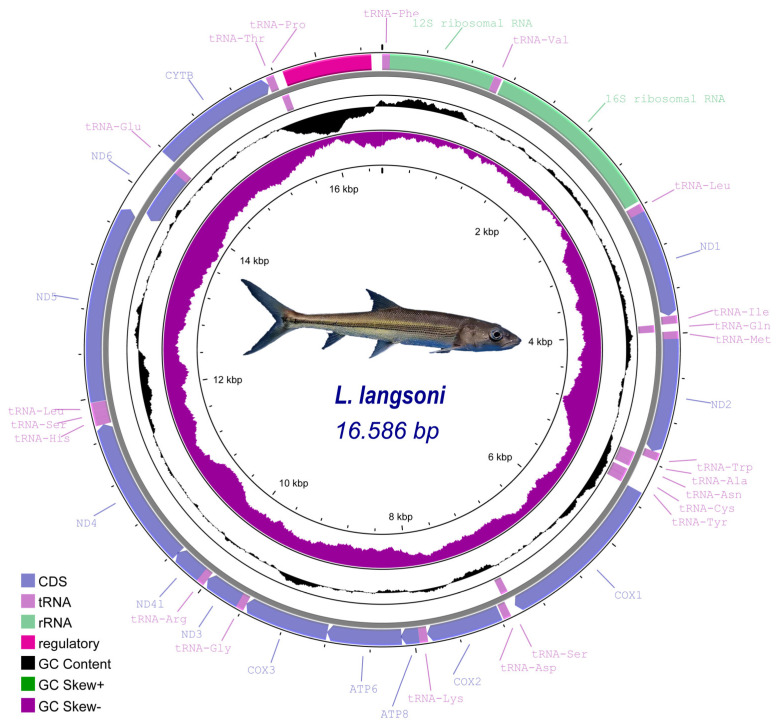
Circular representation of the mitogenome of *L. langsoni*. Image of the *L. langsoni* is shown in the middle. The concentric circles from outermost to innermost illustrate genes located on the heavy chain, genes located on the light chain, GC content (black), and GC skew (purple).

The mitochondrial genome of *L. langsoni* (16,586 bp) was sequenced and annotated in this study, accessible under GenBank Accession No. MZ921933. It is nearly identical in size to that of *L. striolatus* (16,601 bp). This genome is composed of 13 PCGs, two rRNAs, 22 tRNAs, and two control regions (OH, OL), as depicted in Figure 1 and detailed in Table 3. A minority of genes, including *tRNA^Gln^*, *tRNA^Ala^*, *tRNA^Asn^*, *tRNA^Cys^*, *tRNA^Tyr^*, *tRNA^Ser2^*, *tRNA^Glu^*, *tRNA^Pro^*, and *nad6*, are encoded on the light strand, with the remainder on the heavy strand. The gene composition and order in *L. langsoni* are consistent with those typically found in fish mitochondrial genomes [33].

The *L. langsoni* mitochondrial genome features 13 intergenic spacers, ranging from 1 bp to 99 bp in length (Table 3). The longest spacer is situated between OH and *tRNA^Phe^* (99 bp). There are 11 gene overlaps within the genome, with the smallest being 1 bp between OL and *tRNA^Cys^*, *tRNA^Cys^* and *tRNA^Tyr^*, *atp6* and *cox3*, *cox3* and *tRNA^Arg^*, and *tRNA^Th^*^r^ and *tRNA^Pro^* and the largest being 7 bp between *atp8* and *atp6* and *nad4l* and *nad4*. These gene overlaps and spacers are common among most *Cyprininae* fish species.

**Figure 2:**
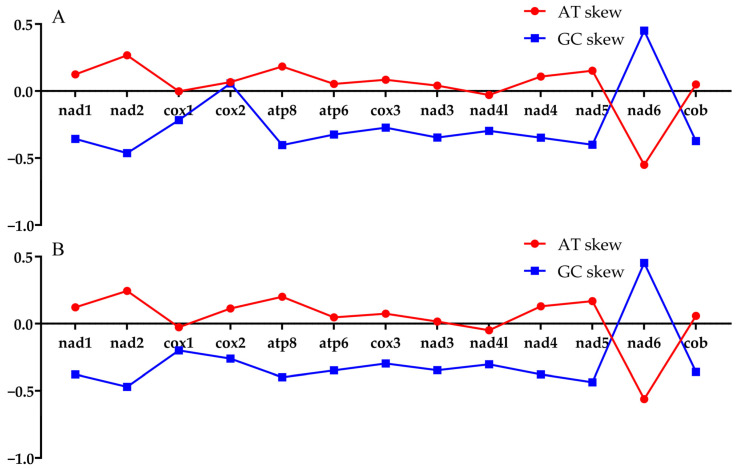
Visualization of the AT and GC skew in the protein-coding genes (PCGs) of *L. langsoni* (**A**) and *L. striolatus* (**B**).

The nucleic acid composition information of the mitochondrial genome of *L. langsoni* is 32.04% A, 24.13% T, 27.88% C, and 15.94% G, exhibiting a gentle AT bias (56.17%) as detailed in Table 4. This composition is similar to other *Cyprininae* fish species. In the non-coding control region sequence, there is the highest A + T content (68.53%), while the lowest is in the first codon position of PCGs (47.94%). The AT skew is positive (0.1407) and the GC skew is negative (−0.2725) in *L. langsoni*, indicating a preference for A and C bases over T and G. Additionally, this study compared the AT and GC skew values of 13 PCGs between two species of *Luciocyprinus*. As shown in Figure 2, the AT skew values are positive for all genes except *cox1* and *nad6*, suggesting an excess of A over T. The greatest difference between the two species is observed in the GC skew of the *cox2* gene, with *L. langsoni* showing a positive value and *L. striolatus* showing a negative one.

### 3.2. Protein-Coding Genes and Codon Usage

The cumulative nucleotide length of the 13 PCGs in *L. langsoni* is 11,382 bp. Most PCGs initiate with the standard ATN start codon, with the exception of the *cox1* gene, which begins with GTG, a recognized canonical mitochondrial start codon for teleosts [20,34,35]. Termination codons in these PCGs predominantly consist of TAA and TAG. Specifically, *nad2*, *atp8*, *nad3*, and *nad6* conclude with TAG, while *nad1*, *cox1*, *atp6*, *cox3*, *nad4l*, and *nad5* end with TAA; the leftover genes, *cox2*, *nad4*, and *cob*, utilize the incomplete T- as their stop codon. This incomplete stop codon, common in metazoan mitogenomes, is hypothesized to be completed through post-transcriptional polyadenylation [36].

**Figure 3:**
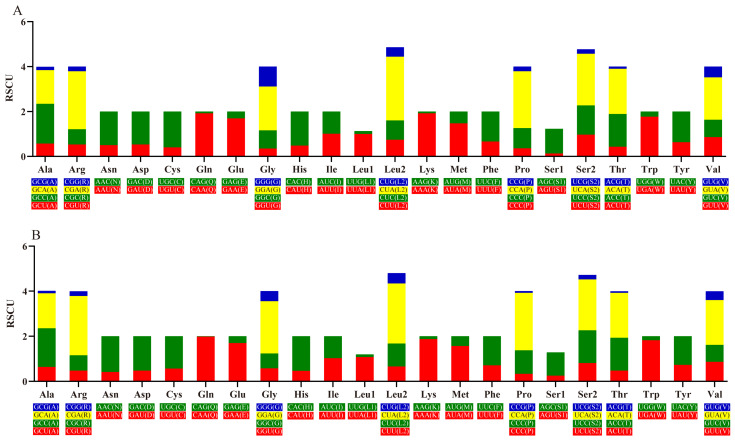
The summary of the RSCU values of PCGs in the mitochondrial genome of *L. langsoni* (**A**) and *L. striolatus* (**B**).

**Figure 4:**
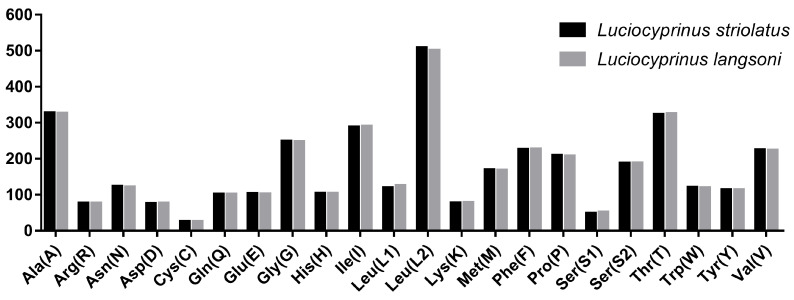
Codon frequency distribution in the mitogenomes of *L. langsoni* and *L. striolatus*. The left side numbers indicate the total count of each codon, with codon families plotted along the X-axis.

Codon usage has important applications in fields such as gene expression optimization, genetic engineering, evolutionary research, bioinformatics, and vaccine design [37]. The summary of the RSCU values of protein-coding sequences in the mitochondrial genome of *L. langsoni* and *L. striolatus* is shown in Figure 3. A grand sum of 3804 triplet codons was used in the 13 PCGs (Table 5). As depicted in Figure 4, the most frequently used amino acids in both *L. langsoni* and *L. striolatus* are Leu, followed by Ala, Thr, Ile, and Gly, with Cys being the least common. In *L. langsoni*, the most common codon is CUA, followed by ACA, UUC, AUU, and GCC, while the least frequent codon, excluding stop codons, is AAG.

### 3.3. Ribosomal and Transfer RNA Genes

In the *L. langsoni* mitogenome, the ribosomal RNA genes, essential for ribosome structure and protein synthesis, include the small (*12S*) and large (*16S*) rRNA genes. These genes, measuring 955 bp and 1641 bp, respectively, are encoded on the heavy strand and situated adjacent to each other, with the *12S rRNA* gene positioned between *tRNA^Phe^* and *tRNA^Val^* and the *16S rRNA* gene positioned between *tRNA^Val^* and *tRNA^Leu2^* (Table 3). The A + T content of these rRNA genes is 54.39%, and their concatenated sequence shows a positive A + T skew of 0.2904 and a negative G + C skew of −0.0980, indicating a compositional bias towards A and T nucleotides over G and C (Table 4).

**Figure 5:**
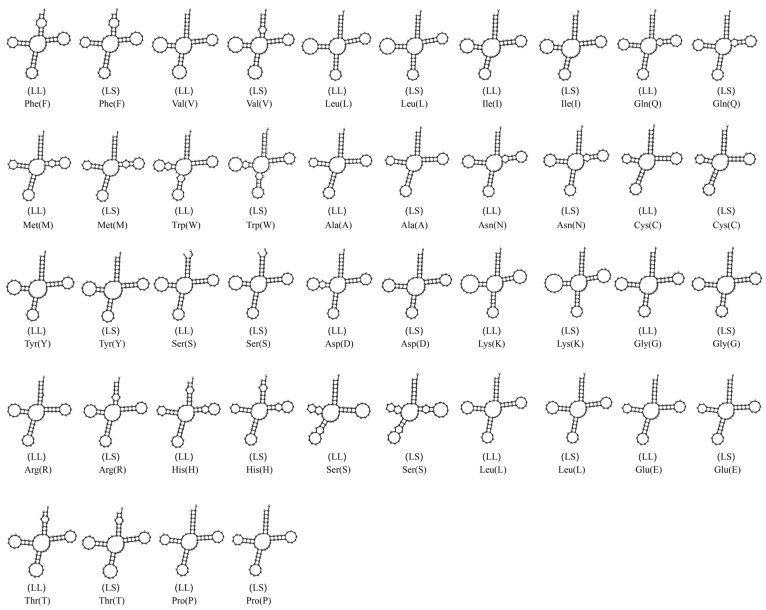
Projected molecular structure for the tRNA genes in the *L. langsoni* (LL) and *L. striolatus* (LS).

In this study, we analyzed and compared the secondary structures of the transfer RNA (tRNA) genes of two species within the genus *Luciocyprinus*, *L. langsoni* and *L. striolatus*. Figure 5 illustrates the secondary structures of the 22 tRNA genes, which varied in size from 67 bp (*tRNA^Cys^*) to 76 bp (*tRNA^Leu2^* and *tRNA^Lys^*). We observed that only tRNASer1 lacked the dihydrouridine (DHU) arm in both species, while the rest formed the typical cloverleaf secondary structure, a feature commonly absent in many fish species [38]. These tRNA genes are distributed across the mitogenome and exhibit a high A + T bias of 54.44%, with a positive A + T skew of 0.0399 and a positive G + C skew of 0.0435 (Table 4). The mitogenome of *L. langsoni* comprises 22 tRNA genes (two for Ser and Leu and one for each of the other amino acids), accounting for 9.4% (1565 bp) of the total genome length. Of these, 15 tRNA genes are encoded on the H strand and 7 are encoded on the L strand (Table 3), a pattern consistent with other *Cyprininae* species [39,40].

### 3.4. Selection Analysis

**Figure 6:**
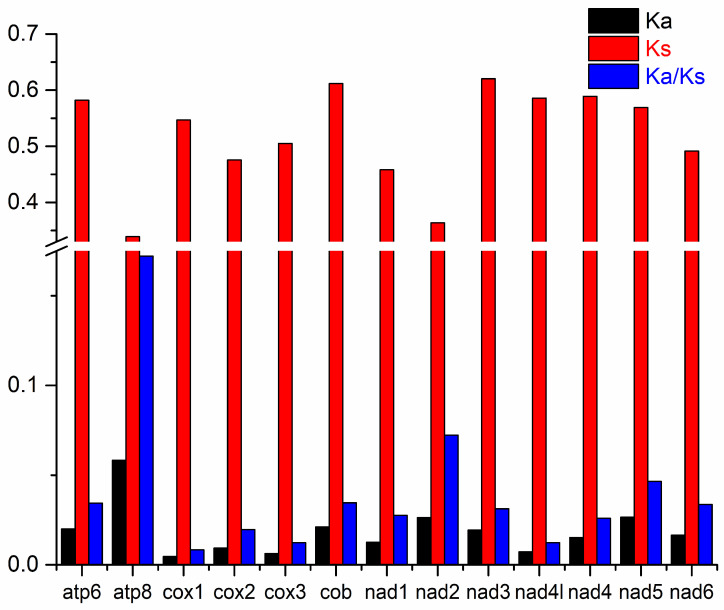
The Ka, Ks, and Ka/Ks values of 13PCGs in *L. langsoni*.

To elucidate the evolutionary dynamics of the 13 PCGs and discern the impact of selective pressures, we determined the Ka, Ks, and Ka/Ks (ω) ratios for each gene (Figure 6). The *cox1* gene exhibited the lowest Ka value of 0.0046, while the *atp8* gene displayed the highest at 0.0584. This is in stark contrast to the analysis results of *L. laticeps*, where the *nad4l* gene had the lowest value at 0.486 and the *nad3* gene had the highest at 0.78. Conversely, *atp8* had the lowest Ks value of 0.3390, and *nad3* possessed the highest at 0.6203, which is also greatly different from the results of *L. laticeps* [34]. The mean ω was 0.0409, ranging from 0.0084 for cox1 to 0.1722 for atp8. Although there are differences in values, the genes with the maximum and minimum ω values are consistent with those of *L. laticeps* and *Hemiculterella wui* [35]. However, different species may show different results. For example, in *Triplophysa labiate* [41], the gene with the smallest ω value is *cox2*, and in *Onychostoma ovale* [20], the gene with the largest ω value is *nad6*. In this paper, these findings suggest that the *atp8* gene may be under more rapid evolutionary pressure compared to other mitochondrial PCGs. Since all PCGs’ ω values are significantly below one, it indicates that these genes have evolved under purifying selection in *L. langsoni* [42].

### 3.5. Phylogenetic Analyses

**Figure 7:**
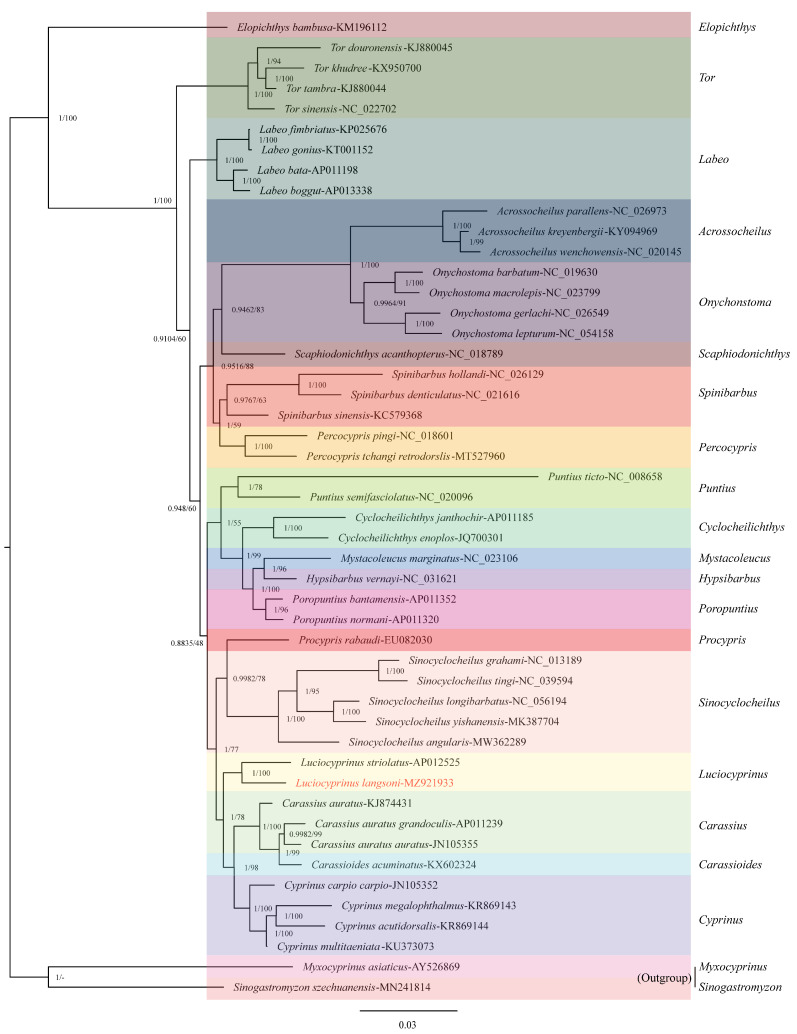
Phylogenetic analyses of *L. langsoni* based on the amino acid sequences of the 13 PCGs within the mitogenome. Branch labels denote posterior probabilities from Bayesian inference (BI) and maximum likelihood (ML) methods. Distinct colors correspond to different genera, “-” indicates that no values are presented.

In this study, we constructed phylogenetic trees utilizing a nucleotide dataset comprising 13 PCGs from 48 species, with *Cyanoplax cavema* and *Nudibranchia californica* as outgroups. The optimal models selected by PartitionFinder were MTMAM + I + G + F for *nad1*, *nad2*, *nad3*, *nad5*, *cox2*, and *atp6*; JTT + I + G + F for *cox1*; MTMAM + G for *atp8*; MTREV + I + G + F for *cox3*; MTMAM + G + F for *nad4l*; MTMAM + I + G for *nad4*; and HIVB + I + G + F for *nad6*. The resulting topologies were largely congruent across different analytical methods (Figure 7). *L. langsoni* and *L. striolatus* clustered together within the genus *Luciocyprinus* with posterior probabilities of 1 and bootstrap proportions of 100%. The phylogeny indicates that *Luciocyprinus*, along with *Cyprinus*, *Carassioides*, and *Carassius*, form a larger clade while being more distantly related to the morphologically similar *Elopichthys*. Our findings support the taxonomic classification of *Luciocyprinus* within the subfamily *Cyprininae* rather than *Barbinae* [14]. However, due to limited research on *Luciocyprinus*, further studies are needed to refine its specific taxonomic affiliations.

### 3.6. Population Genetic Diversity

Genetic diversity underpins a species’ survival, evolution, and adaptability to environmental changes; higher genetic diversity correlates with greater adaptability and evolutionary potential. The *COI* gene sequence, known for its high genetic polymorphism, has been widely utilized in genetic diversity studies of various fish species [43]. In this study, we obtained the majority of the *COI* gene sequence from *L. langsoni*, yielding a total length of 1664 base pairs (bp) after alignment and refinement, with only two distinct haplotypes identified. Within this research, the population of *L. langsoni* from Hechi Longjiang exhibited a haplotype diversity (Hd) of 0.497 and a nucleotide diversity (π) of 0.0003, indicating low haplotype diversity (Hd < 0.5) and low nucleotide diversity (π < 0.005) (Table 6). These findings suggest that the population may have undergone a genetic bottleneck, implying a significant reduction in population size in the past with few founding individuals, leading to reduced genetic diversity [44]. This is further supported by the positive value of Tajima’s D for the Hechi Longjiang population, indicating a sudden population size reduction followed by a subsequent recovery [45].

## 4. Conclusions

We successfully determined the mitogenome sequence of *L. langsoni* through overlapping PCR, which spanned 16,586 bp and encompassed 13 PCGs, 22 tRNA genes, 2 rRNA genes, and two control regions (OH, OL). This gene arrangement and nucleotide composition, with a marked A + T bias, are consistent with those reported in other teleosts. The majority of PCGs initiate with the ATG start codon and terminate with the TAA stop codon. Ka/Ks ratio analysis revealed that all 13 PCGs in *L. langsoni* have evolved under purifying selection, with ratios all below 1. These findings, along with the phylogenetic trees, support the classification of *Luciocyprinus* within the subfamily *Cyprininae* rather than *Barbinae*. The low genetic diversity observed in the Hechi Longjiang population, based on *COI* gene analysis, suggests the population may have experienced a genetic bottleneck. This study’s results enhance our understanding of phylogenetic relationships and provide foundational genetic data for future population genetic studies and phylogenetic analysis of *L. langsoni* and related genera.

Due to its large body size and beautiful morphology, *L. langsoni* is a species with great aquaculture prospects. The mitochondrial genome obtained in this study, along with the result of low population genetic diversity, can assist in analyzing the kinship between individuals in the formulation of breeding strategies in the aquaculture industry [46]. For example, in the process of selecting the parents of *L. langsoni*, it is appropriate to choose individuals from different river systems or those with distant kinship. Additionally, some genes in the mitochondrial genome, such as the *COI* gene, are often used in environmental DNA analysis. For instance, the *COI* gene is commonly employed as a template for designing environmental DNA amplification primers to identify species diversity in a certain area or the presence of a certain species [47,48]. The mitochondrial genome obtained in this paper can serve as a primer design template, enabling the detection of whether *L. langsoni* exists in different rivers by using environmental DNA detection technology. It can also be applied to conduct process evaluations for the future stock enhancement and release of *L. langsoni* and the restoration of its biological community.

## Figures and Tables

**Table 1:** Twelve pairs of PCR primers designed in this study for amplifying the full-length mitochondrial genome of *L. langsoni*.

Primer Name	Primer Sequences (5′-3′)	Annealing Temperature
LL-F1	ACATGCAAGTCTCCGCAACC	57.9 °C
LL-R1	TTGGCTTACACTTGTGCTTGGA	
LL-F2	CAGCCACCTAAACAGAAAGCG	55.8 °C
LL-R2	GGGCGATGTTAAATGTTTGTAG	
LL-F3	TCCTATTTATCCTAGCCCTGTC	52.8 °C
LL-R3	AATCCTGTGAGTGGTGGTAGG	
LL-F4	CGCAGCATTCCTAACCCTAA	54.4 °C
LL-R4	GGATGTAAAGTATGCACGAGTGT	
LL-F5	CACCACATTCTTCGACCCG	56.2 °C
LL-R5	TCCTAGCGAGGCGTCTTCTAG	
LL-F6	CGACTAAATCAAACCGCCTTCA	58.2 °C
LL-R6	CTTGTTTGCGTTCTCCTTCCA	
LL-F7	CCAACACCAGAATTAGGAGGAT	54.6 °C
LL-R7	CATTGAGTCGTTCGGTTTGAT	
LL-F8	ATACATCTCACTCCTTGCCTCA	51.4 °C
LL-R8	TTATGGCTACTAGGAATGTGAG	
LL-F9	CTGACAATGAATAAACACCCAAAC	55.5 °C
LL-R9	TCGTGCGGTTTAGAGGAGG	
LL-F10	ATCAGCCGCCACCCAACT	58.1 °C
LL-R10	TTGGGATTGATCGTAGGATTGC	
LL-F11	TTTCCACCCATACTTCTCATA	49.9 °C
LL-R11	AGCGGTTTGGTGATAATACA	
LL-F12	CTCCCTAGCGCCCAGAAA	54.9 °C
LL-F12	AGGGTTTCGGGCACCTAG	

**Table 2:** Detailed species information of mitochondrial genomes used for evolutionary relationship analysis in this paper.

Genus	Species	Length (bp)	GenBank Accession Number
*Elopichthys*	*Elopichthys bambusa*	16,619	KM196112
*Luciocyprinus*	*L. langsoni*	16,586	MZ921933 (this study)
	*L. striolatus*	16,601	AP012525
*Cyprinus*	*Cyprinus multitaeniata*	16,573	KU373073
	*Cyprinus carpio carpio*	16,581	JN105352
	*Cyprinus acutidorsalis*	16,580	KR869144
	*Cyprinus megalophthalmus*	16,580	KR869143
*Procypris*	*Procypris rabaudi*	16,595	EU082030
*Carassioides*	*Carassioides acuminatus*	16,579	KX602324
*Carassius*	*Carassius auratus*	16,576	KJ874431
	*C. auratus auratus*	16,580	JN105355
	*C. auratus grandoculis*	16,579	AP011239
*Labeo*	*Labeo bata*	16,605	AP011198
	*Labeo boggut*	16,603	AP013338
	*Labeo fimbriatus*	16,614	KP025676
	*Labeo gonius*	16,614	KT001152
*Poropuntius*	*Poropuntius bantamensis*	16,594	AP011352
	*Poropuntius normani*	16,592	AP011320
*Cyclocheilichthys*	*Cyclocheilichthys janthochir*	16,580	AP011185
	*Cyclocheilichthys enoplos*	16,579	JQ700301
*Onychonstoma*	*Onychostoma barbatum*	16,592	NC_019630
	*Onychostoma gerlachi*	16,601	NC_026549
	*Onychostoma lepturum*	16,601	NC_054158
	*Onychostoma macrolepis*	16,595	NC_023799
*Spinibarbus*	*Spinibarbus sinensis*	16,591	KC579368
	*Spinibarbus denticulatus*	16,549	NC_021616
	*Spinibarbus hollandi*	16,521	NC_026129
*Acrossocheilus*	*Acrossocheilus kreyenbergii*	16,596	KY094969
	*Acrossocheilus parallens*	16,592	NC_026973
	*Acrossocheilus wenchowensis*	16,591	NC_020145
*Tor*	*Tor douronensis*	16,586	KJ880045
	*Tor khudree*	16,576	KX950700
	*Tor sinensis*	16,579	NC_022702
	*Tor tambra*	16,581	KJ880044
*Sinocyclocheilus*	*Sinocyclocheilus angularis*	16,586	MW362289
	*Sinocyclocheilus grahami*	16,585	NC_013189
	*Sinocyclocheilus longibarbatus*	16,787	NC_056194
	*Sinocyclocheilus tingi*	16,584	NC_039594
	*Sinocyclocheilus yishanensis*	16,573	MK387704
*Percocypris*	*Percocypris pingi*	16,586	NC_018601
	*Percocypris tchangi retrodorslis*	16,576	MT527960
*Hypsibarbus*	*Hypsibarbus vernayi*	16,590	NC_031621
*Puntius*	*Puntius semifasciolatus*	16,594	NC_020096
	*Puntius ticto*	17,302	NC_008658
*Mystacoleucus*	*Mystacoleucus marginatus*	16,611	NC_023106
*Scaphiodonichthys*	*Scaphiodonichthys acanthopterus*	16,612	NC_018789
*Sinogastromyzon*	*S. szechuanensis*	16,565	MN241814
*Myxocyprinus*	*M. asiaticus*	16,636	AY526869

**Table 3:** The gene characteristic information of the mitochondrial genome of *L. langsoni*. H: heavy chain; L: light chain.

Name	Strand	Location	Size (bp)	Intergenic Length	Anti-Codon	Start Codon	Stop Codon
*tRNA^Phe^*	H	1–69	69	0	GAA	-	-
*12S rRNA*	H	70–1024	955	2	-	-	-
*tRNA^Val^*	H	1027–1098	72	18	TAC	-	-
*16S rRNA*	H	1117–2757	1641	25	-	-	-
*tRNA^Leu2^*	H	2783–2858	76	1	TAA	-	-
*nad1*	H	2860–3834	975	4	-	ATG	TAA
*tRNA^Ile^*	H	3839–3910	72	−2	GAT	-	-
*tRNA^Gln^*	L	3909–3979	71	1	TTG	-	-
*tRNA^Met^*	H	3981–4049	69	0	CAT	-	-
*nad2*	H	4050–5096	1047	−2	-	ATG	TAG
*tRNA^Trp^*	H	5095–5165	71	2	TCA	-	-
*tRNA^Ala^*	L	5168–5236	69	1	TGC	-	-
*tRNA^Asn^*	L	5238–5310	73	2	GTT	-	-
*OL*	H	5313–5344	32	−1	-	-	-
*tRNA^Cys^*	L	5344–5410	67	−1	GCA	-	-
*tRNA^Tyr^*	L	5410–5480	71	1	GTA	-	-
*cox1*	H	5482–7032	1551	0	-	GTG	TAA
*tRNA^Ser2^*	L	7033–7103	71	3	TGA	-	-
*tRNA^Asp^*	H	7107–7178	72	13	GTC	-	-
*cox2*	H	7192–7882	691	0	-	ATG	T(AA)
*tRNA^Lys^*	H	7883–7958	76	1	TTT	-	-
*atp8*	H	7960–8124	165	−7	-	ATG	TAG
*atp6*	H	8118–8801	684	−1	-	ATG	TAA
*cox3*	H	8801–9586	786	−1	-	ATG	TAA
*tRNA^Gly^*	H	9586–9657	72	0	TCC	-	-
*nad3*	H	9658–10,008	351	−2	-	ATG	TAG
*tRNA^Arg^*	H	10,007–10,077	71	0	TCG	-	-
*nad4l*	H	10,078–10,374	297	−7	-	ATG	TAA
*nad4*	H	10,368–11,748	1381	0	-	ATG	T(AA)
*tRNA^His^*	H	11,749–11,817	69	0	GTG	-	-
*tRNA^Ser1^*	H	11,818–11,886	69	1	GCT	-	-
*tRNA^Leu1^*	H	11,888–11,960	73	3	TAG	-	-
*nad5*	H	11,964–13,787	1824	−4	-	ATG	TAA
*nad6*	L	13,784–14,305	522	0	-	ATG	TAG
*tRNA^Glu^*	L	14,306–14,374	69	5	TTC	-	-
*cob*	H	14,380–15,520	1141	0	-	ATG	T(AA)
*tRNA^Thr^*	H	15,521–15,593	73	−1	TGT	-	-
*tRNA^Pro^*	L	15,593–15,662	70	18	TGG	-	-
*OH*	H	15,681–16,487	807	99	-	-	-

**Table 4:** Table of nucleic acid composition and skewness information of the mitochondrial genome of *L. langsoni*.

Location	Size (bp)	A	T	G	C	A + T	G + C	A + T Skew	G + C Skew
Genome	16,586	32.04	24.13	15.94	27.88	56.17	43.83	0.1407	−0.2725
PCGs	11,382	29.85	26.03	15.45	28.66	55.89	44.11	0.0684	−0.2993
1st codon position	3794	27.10	20.85	25.75	26.30	47.94	52.06	0.1303	−0.0106
2nd codon position	3794	18.37	40.43	13.63	27.57	58.80	41.20	−0.3752	−0.3385
3rd codon position	3794	44.10	16.82	6.98	32.10	60.91	39.09	0.4479	−0.6426
rRNA	2596	35.09	19.30	20.57	25.04	54.39	45.61	0.2904	−0.0980
tRNA	1565	28.31	26.13	23.77	21.79	54.44	45.56	0.0399	0.0435
control region	807	35.19	33.33	13.63	17.84	68.53	31.47	0.0271	−0.1339

**Table 5:** Codon number and RSCU of mitochondrial PCGs in *L. langsoni*.

Amino Acid	Codon	Count	RSCU	Amino Acid	Codon	Count	RSCU
Phe	UUU	75	0.66	Tyr	UAU	36	0.63
Phe	UUC	151	1.34	Tyr	UAC	78	1.37
Leu	UUA	105	1	stop codon	UAA	6	2.4
Leu	UUG	14	0.13	stop codon	UAG	4	1.6
Leu	CUU	77	0.74	His	CAU	25	0.48
Leu	CUC	90	0.86	His	CAC	79	1.52
Leu	CUA	297	2.84	Gln	CAA	97	1.92
Leu	CUG	44	0.42	Gln	CAG	4	0.08
Ile	AUU	145	1.01	Asn	AAU	31	0.5
Ile	AUC	143	0.99	Asn	AAC	92	1.5
Met	AUA	124	1.47	Lys	AAA	74	1.92
Met	AUG	45	0.53	Lys	AAG	3	0.08
Val	GUU	48	0.86	Asp	GAU	20	0.53
Val	GUC	43	0.77	Asp	GAC	55	1.47
Val	GUA	106	1.89	Glu	GAA	87	1.69
Val	GUG	27	0.48	Glu	GAG	16	0.31
Ser	UCU	38	0.97	Cys	UGU	5	0.4
Ser	UCC	51	1.3	Cys	UGC	20	1.6
Ser	UCA	90	2.3	Trp	UGA	106	1.77
Ser	UCG	8	0.2	Trp	UGG	14	0.23
Pro	CCU	19	0.36	Arg	CGU	10	0.53
Pro	CCC	47	0.9	Arg	CGC	13	0.68
Pro	CCA	132	2.53	Arg	CGA	49	2.58
Pro	CCG	11	0.21	Arg	CGG	4	0.21
Thr	ACU	35	0.43	Ser	AGU	5	0.13
Thr	ACC	118	1.46	Ser	AGC	43	1.1
Thr	ACA	162	2.01	stop codon	AGA	0	0
Thr	ACG	8	0.1	stop codon	AGG	0	0
Ala	GCU	47	0.57	Gly	GGU	22	0.35
Ala	GCC	145	1.77	Gly	GGC	50	0.81
Ala	GCA	123	1.5	Gly	GGA	121	1.95
Ala	GCG	12	0.15	Gly	GGG	55	0.89

**Table 6:** Genetic diversity parameters of *COI* gene in Hechi Longjiang population.

Population	Number of Haplotypes	Haplotype (Gene) Diversity	Average Number of Nucleotide Difference	Nucleotide Diversity	Tajima’s D
Hechi Longjiang	2	0.497	0.497	0.0003	1.5078

## Data Availability

The mitochondrial DNA sequence generated from the sample has been deposited in the GenBank database (https://www.ncbi.nlm.nih.gov, 13 September 2022) with the accession number MZ921933.
